# Crystal structure, Hirshfeld surface analysis and DFT studies of 1-[*r*-2,*c*-6-diphenyl-*t*-3-(propan-2-yl)piperidin-1-yl]ethan-1-one

**DOI:** 10.1107/S2056989020002042

**Published:** 2020-02-18

**Authors:** P. Periyannan, M. Beemarao, K. Karthik, S. Ponnuswamy, K. Ravichandran

**Affiliations:** a Department of Physics, Kandaswami Kandar’s College, Velur, Namakkal 638 182, India; b PG and Research Department of Chemistry, Government Arts College (Autonomous), Coimbatore 641 018., Tamil Nadu, India

**Keywords:** crystal structure, piperidine derivative, Hirshfeld surface, DFT

## Abstract

In the title compound, C_22_H_27_NO, the piperidine ring adopts a chair conformation. The dihedral angles between the mean plane of the piperidine ring and the phenyl rings are 89.78 (7) and 48.30 (8)°. In the crystal, mol­ecules are linked into chains along the *b*-axis direction by C—H⋯O hydrogen bonds. The DFT/B3LYP/6–311 G(d,p) method was used to determine the HOMO–LUMO energy levels. The mol­ecular electrostatic potential surfaces were investigated by Hirshfeld surface analysis and two-dimensional fingerprint plots were used to analyse the inter­molecular inter­actions in the mol­ecule.

## Abstract 2

The dihedral angles between the mean plane of the piperidine ring, which adopts a chair conformation, and the phenyl rings are 89.72 (8) and 48.32 (8)°. In the crystal, mol­ecules are linked into chains along the *b*-axis direction by C—H⋯O hydrogen bonds.

## Chemical context   

Piperidine is a heterocyclic six-membered ring containing nitro­gen as a hetero atom and is an essential structural part of many important drugs including paroxetine, raloxifene, haloperidol, droperidol and minoxidiln (Wagstaff *et al.*, 2002 ▸). Piperidine derivatives exhibit a wide range of biological activities, such as anti­microbial, anti-inflammatory, anti­viral, anti­malarial and general anesthetic (Aridoss *et al.*, 2009 ▸). The biological properties of piperidines are highly dependent on the type and position of substituents on the heterocyclic ring. 2,6-Disubstituted piperidine derivatives have been found to possess fungicidal, bactericidal and herbicidal activities (Mobio *et al.*, 1989 ▸). Piperidine derivatives are the inter­mediate products in agrochemicals, pharmaceuticals, rubber vulcanization accelerators and are widely used as building block mol­ecules in many industries. Various piperidine derivatives are present in numerous alkaloids (Badorrey *et al.*, 1999 ▸).
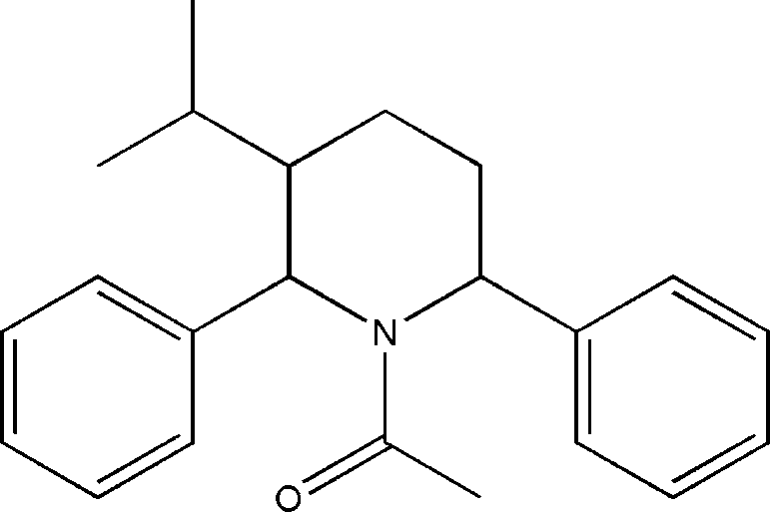


This wide range of biological activities prompted us to synthesize novel 2,6-diphenyl piperdine derivatives. Against this background, the structure of the title compound has been determined.

## Structural commentary   

**Figure 1:**
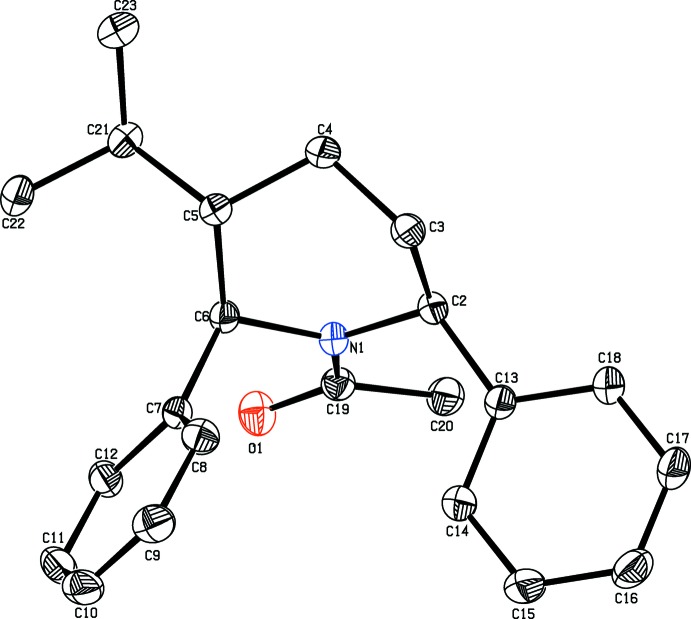
The mol­ecular structure of the title compound, showing the atomic numbering and displacement ellipsoids drawn at the 30% probability level.

The mol­ecular structure of the title compound is shown in Fig. 1 ▸. The diphenyl-substituted piperidine compound crystallizes in the monoclinic space group *P*2_1_/*n*. The bond lengths and angles are well within the expected limits and comparable with literature values (Allen *et al.*, 1998 ▸).

The piperidine ring adopts a chair conformation with the puckering parameters *Q*_2_ = 0.6191 (15) Å and ϕ2 = 335.12 (14) Å. The piperidine ring (N1/C2–C6) makes dihed­ral angles of 89.78 (7) and 48.30 (8)°, respectively, with the C7–12 and C13–C18 phenyl rings, and confirms the fact that the moieties are in an axial orientations.

The keto and methyl groups substituted at atom C19 are equatorially orientated as confirmed from the torsion angle values O1—C19—N1—C2 = 177.54 (12)° and C20—C19—N1—C6 = 172.81 (11)°. In the mol­ecule, the isopropyl group substituted at the 5-position of the piperidine ring is equatorially oriented, as confirmed by the torsion angles of C4—C5—C21—C22 = −172.13 (14)° and C6—C5—C21—C23 = −174.73 (14)°. The sum of the bond angles (359.87°) around atom N1 of the piperidine ring is in accordance with the *sp* ^2^-hybridization state (Beddoes *et al.*, 1986 ▸).

## Supra­molecular features   

**Figure 2:**
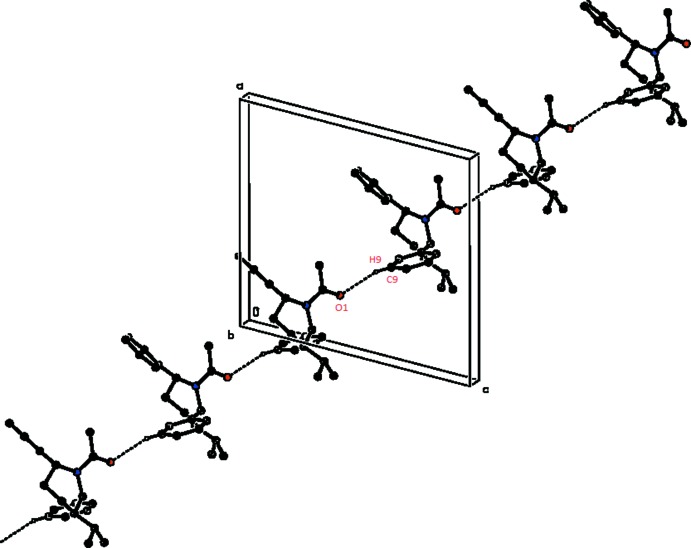
A partial view along the *b* axis of the crystal packing of the title compound, showing the formation of a mol­ecular chain by C—H⋯O inter­actions (dotted lines).

**Figure 3:**
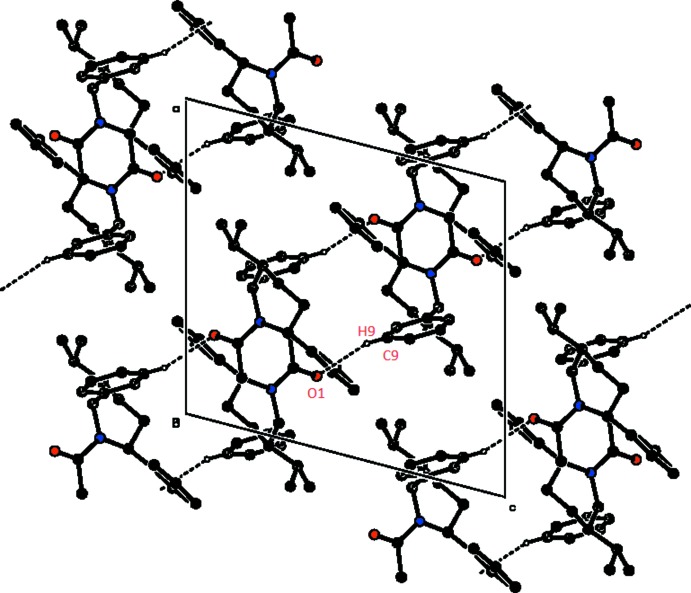
The overall crystal packing of the title compound, viewed along the *b-*axis direction. Hydrogen bonds are shown as dashed lines, and only the H atoms involved in hydrogen bonding have been included.

In the crystal, mol­ecules are linked into *C*(8) chains along the *b*-axis direction by C—H⋯O hydrogen bonds (Table 1 ▸, Fig. 2 ▸). The overall crystal packing of the title compound is shown in Fig. 3 ▸.

## DFT study   

The optimized structure of the mol­ecule in the gas phase was generated theoretically *via* density functional theory (DFT) using standard B3LYP functional and 6-311G(d,p) basis-set calculations (Becke *et al.*, 1993 ▸), as implemented in *GAUSSIAN09* (Frisch *et al.*, 2009 ▸).

**Figure 4:**
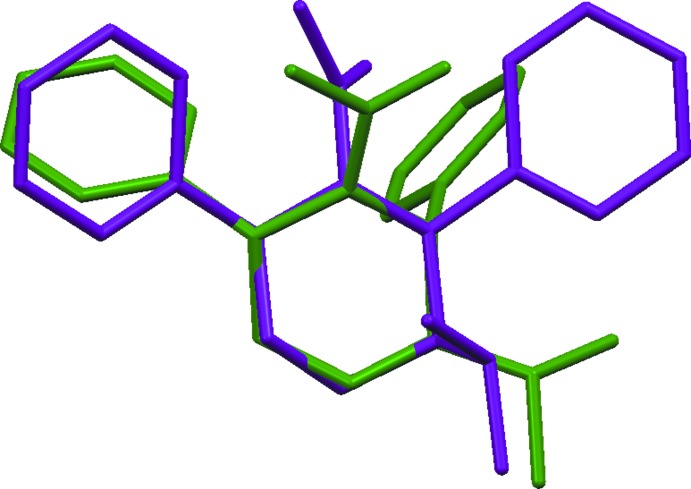
A structural overlay diagram (*Mercury*; Macrae *et al.*, 2020 ▸) for the optimized structure (purple) and the solid-state structure (green) of the title compound.

The overlay diagram for the optimized structure (purple) and the structure in solid state (green) with respect to the piperidine ring is shown in Fig. 4 ▸. The piperidine rings in the two phases have an r.m.s deviation of 0.434 Å for the non-hydrogen atoms. The conformation of the mol­ecules in the two phases differs with respect to the central piperidine ring, as seen in the disparity of about 38.5° in the N1—C6—C5—C4 torsion angles (39.88/1.38°) and 2.25° in the N1—C2—C3—C4 torsion angles (44.41/39.81°) for the optimized and solid-state mol­ecules, respectively.

**Figure 5:**
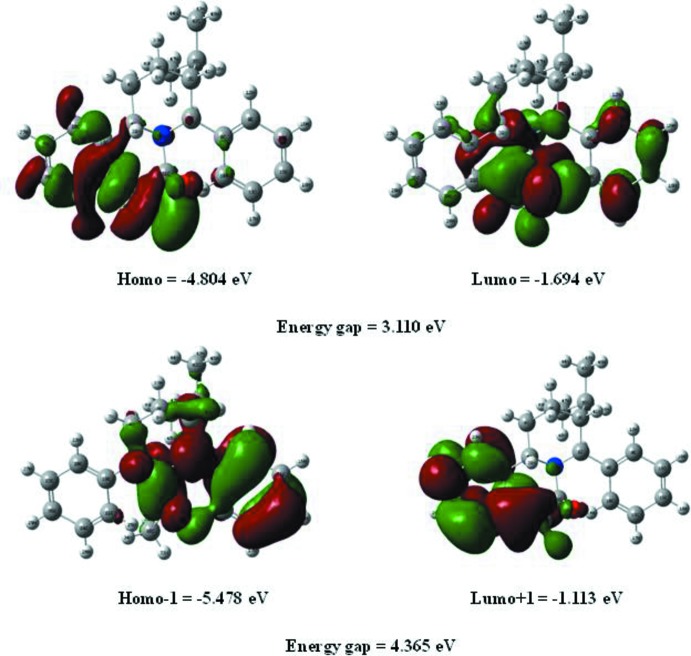
The frontier mol­ecular orbitals (FMOs) of the title compound.

The highest-occupied mol­ecular orbital (HOMO), acting as an electron donor, and the lowest-unoccupied mol­ecular orbital (LUMO), acting as an electron acceptor, are known as frontier mol­ecular orbitals (FMOs). The FMOs play an important role in the optical and electric properties, as well as in quantum chemistry (Fleming, 1976 ▸). When the energy gap is small, the mol­ecule is highly polarizable and has high chemical reactivity. The electron distribution of the HOMO−1, HOMO, LUMO and LUMO+1 energy levels and the energy values are shown in Fig. 5 ▸. The positive and negative phases are shown in green and red, respectively.

The HOMO of the title mol­ecule is localized on the C=O group, one aromatic ring and the piperidine ring, while the LUMO is located over the whole mol­ecule expect for the isopropyl group. The DFT study shows that the FMO energies *E*_HOMO_ and *E*_LUMO_ are −4.804 and −1.694 eV, respectively, and the HOMO–LUMO energy gap is 3.110 eV. The title compound has a small frontier orbital gap, hence the mol­ecule has high chemical reactivity and low kinetic stability.

The electron affinity (*I*) and ionization potential (*A*) of the mol­ecule were calculated using the DFT/B3LYP/6-311++G(d,p) basis set. A high value of the electrophilicity index describes a good electrophile, while a small value of electrophilicity index describes a good nucleophile. The values of the hardness (η), softness (σ), electronegativity (χ) and electrophilicity index (ω) for the title compound are given in Table 2 ▸.

## Hirshfeld surface analysis   

**Figure 6:**
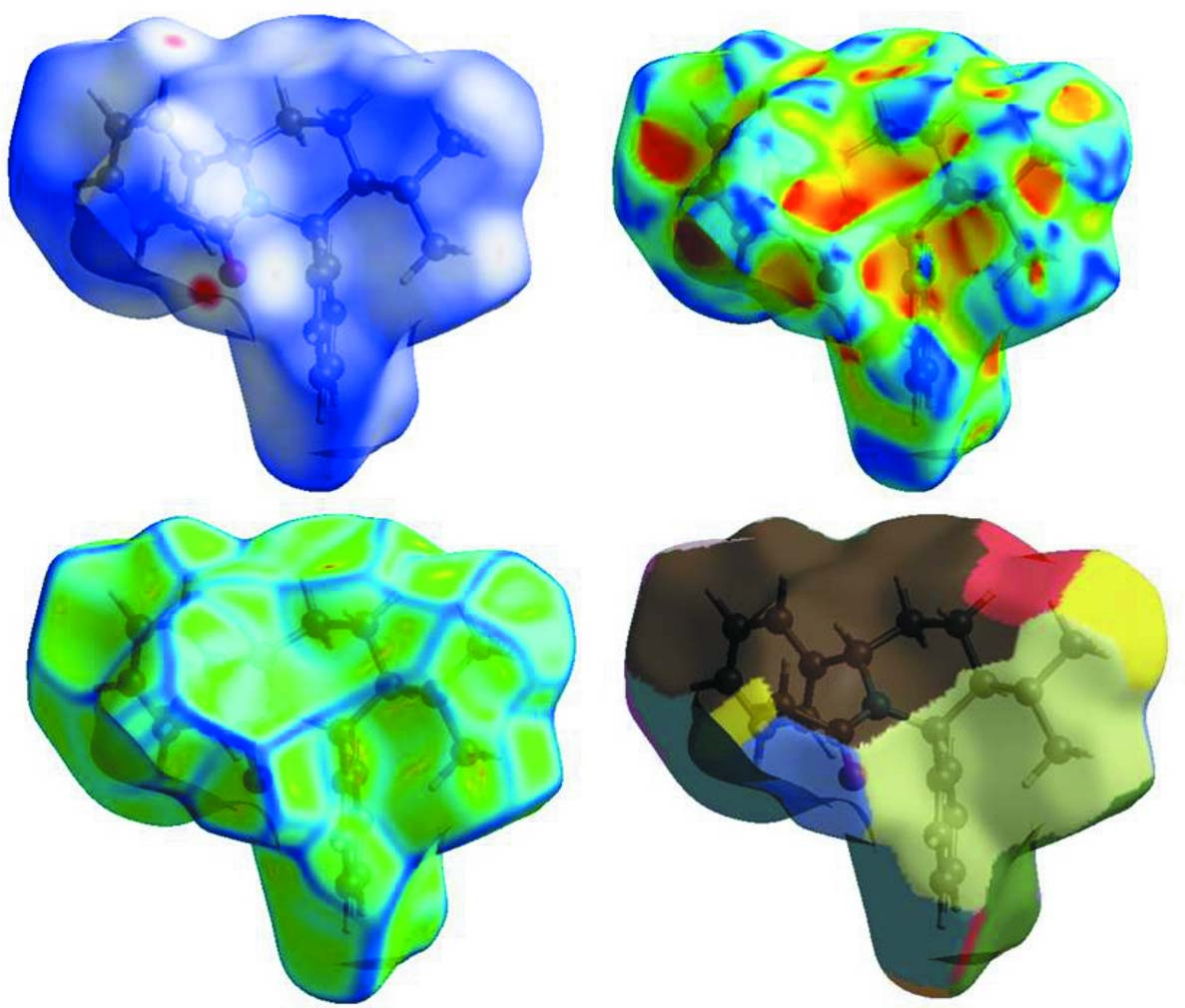
Hirshfeld surfaces mapped over (*a*) *d*_norm_, (*b*) shape-index, (*c*) curvedness and (*d*) fragment patches.

*CrystalExplorer17* (Turner *et al.*, 2017 ▸) was used for the Hirshfeld surface (HS) analysis (Spackman & Jayatilaka, 2009 ▸) and to generate the associated two-dimensional fingerprint plots (McKinnon *et al.*, 2007 ▸) to qu­antify the various inter­molecular inter­actions in the structure of the title compound. In the HS plotted over *d*_norm_ (Fig. 6 ▸), the white surface indicates contacts with distances equal to the sum of the van der Waals radii, and the red and blue colours indicate distances shorter (in close contact) or longer (distinct contact) than the van der Waals radii, respectively (Venkatesan *et al.*, 2016 ▸).

The HS mapped over curvedness and shape-index, introduced by Koendrink (Koenderink, 1990 ▸; Koenderink & van Doorn, 1992 ▸), give further chemical insight into mol­ecular packing. A surface with low curvedness designates a flat region and may be indicative of π–π stacking in the crystal. A Hirshfeld surface with high curvedness is highlighted as dark-blue edges, and is indicative of the absence of π–π stacking (Fig. 6 ▸). The nearest neighbour coordination environment of a mol­ecule is identified from the colour patches on the Hirshfeld surface, depending on their closeness to adjacent mol­ecules (Mohamooda Sumaya *et al.*, 2018 ▸).

**Figure 7:**
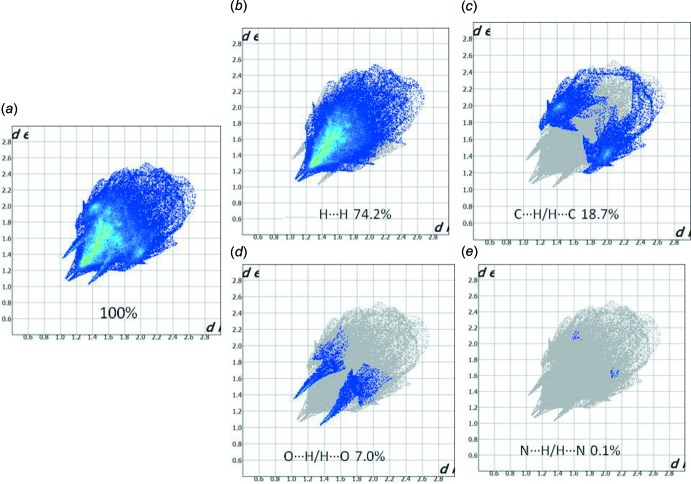
Two-dimensional fingerprint plot for the title compound showing the contributions of individual types of inter­actions: (*a*) all inter­molecular contacts, (*b*) H⋯H contacts, (*c*) C⋯H/H⋯C contacts, (*d*) O⋯H/H⋯O contacts, (*e*) N⋯H/H⋯N contacts.

The 2D fingerprint plots of the *d*_i_ and *d*_e_ points for the contacts contributing to the Hirshfeld surface are shown in Fig. 7 ▸. They indicate that inter­molecular H⋯H contacts provide the largest contribution (74.2%) to the Hirshfeld surface. The percentage contributions of the other inter­actions are C⋯H/H⋯C = 18.7%, O⋯H/H⋯O = 7.0% and N⋯H/H⋯N = 0.1%. The Hirshfeld surface analysis confirms the importance of H-atom contacts in establishing the packing. The large number of H⋯H, H⋯C/C⋯H, H⋯O/O⋯H and H⋯N/N⋯H inter­actions suggest that hydrogen bonding and van der Waals inter­actions play the major roles in the crystal packing (Hathwar *et al.*, 2015 ▸).

## Database survey   

A search of the Cambridge Structural Database (CSD, version 5.39; Groom *et al.*, 2016 ▸) using piperidine as the main skeleton revealed the presence of more than 30 records with different substituents on the piperidine ring. However, there are only two compounds with the same skeleton as the title compound, *viz. r*-2,*c*-6-di­phenyl­piperidine (NIKYEN; Maheshwaran *et al.*, 2013 ▸) and methyl 4-oxo-*r*-2,*c*-6-di­phenyl­piperidine-3-carboxyl­ate (BIHZEY; Sampath *et al.*, 2004 ▸). In these compounds, the piperidine ring adopts a chair conformation as the title compound. The phenyl rings substituted at the 2- and 6-positions of the piperidine ring subtend dihedral angles of 89.78 (7) and 48.30 (8)°, respectively, with the best plane of the piperidine ring in the title compound and 81.04 (7) and 81.10 (7)°, respectively, in NIKYEN, whereas in BIHZEY they are equatorially oriented. The C—H⋯O inter­action leads to the formation of a *C*(8) chain in the title compound, while it forms dimers in the other two structures.

## Synthesis and crystallization   

*t*-3-Isopropyl-*r*-2,*c*-6-di­phenyl­piperidin-4-one was reduced to the corresponding piperidine using the Wolf–Kishner reduction (Ravindran & Jeyaraman, 1992 ▸). Piperidine-4-one (10 mmol) was treated with di­ethyl­ene glycol (40 ml), hydrazine hydrate (10 mmol) and KOH pellets (10 mmol) to give *t*-3-isopropyl-*r*-2,*c*-6-di­phenyl­piperidine. *N*-Acetyl piperidine was synthesized by the acetyl­ation of the above piperidine. To *t*-3-isopropyl-*r*-2,*c*-6-di­phenyl­piperidine (5 mmol) dissolved in benzene (50 ml) were added tri­ethyl­amine (20 mmol) and acetyl chloride (20 mmol) to give the title compound, which was crystallized by slow evaporation from a benzene/petroleum ether **(** ***v*** **:** ***v*** **= ?:?)** solution.

## Refinement   

Crystal data, data collection and structure refinement details are summarized in Table 3 ▸. H atoms were positioned geometrically (N—H = 0.88–0.90 Å and C—H = 0.93–0.98 Å) and allowed to ride on their parent atoms,with *U*_iso_(H) = 1.5*U*eq(C) for methyl H 1.2*U*eq(C) for other H atoms.

## Supplementary Material

Crystal structure: contains datablock(s) global, I. DOI: 10.1107/S2056989020002042/dx2023sup1.cif

Structure factors: contains datablock(s) I. DOI: 10.1107/S2056989020002042/dx2023Isup2.hkl

Supporting information file. DOI: 10.1107/S2056989020002042/dx2023Isup3.cml

CCDC reference: 1814839

Additional supporting information:  crystallographic information; 3D view; checkCIF report

## Figures and Tables

**Table 1:** Hydrogen-bond geometry (Å, °)

*D*—H⋯*A*	*D*—H	H⋯*A*	*D*⋯*A*	*D*—H⋯*A*
C9—H9⋯O1^i^	0.93	2.54	3.4378 (19)	163

Symmetry code: (i) 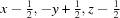.

**Table 2:** Calculated frontier mol­ecular orbital analysis of the title compound

Parameter	Value
E_HOMO_ (eV)	−4.804
E_LUMO_ (eV)	−1.694
Energy gap, Δ*E* (eV)	3.110
HOMO−1 (eV)	−5.478
LUMO+1 (eV)	−1.113
Ionization potential, *I* (eV)	4.804
Electron affinity, *A*	1.694
Electrophilicity Index, ω	3.394
Hardness, η	1.555
Electro negativity, χ	3.249
Softness, σ	0.322

**Table 3:** Experimental details

Crystal data	
Chemical formula	C_22_H_27_NO
*M*_r_	321.44
Crystal system, space group	Monoclinic, *P*2_1_/*n*
Temperature (K)	296
*a*, *b*, *c* (Å)	13.3077 (5), 10.3009 (4), 13.9338 (5)
β (°)	104.657 (1)
*V* (Å^3^)	1847.91 (12)
*Z*	4
Radiation type	Mo *K*α
μ (mm^−1^)	0.07
Crystal size (mm)	0.30 × 0.25 × 0.20

Data collection	
Diffractometer	Bruker *SMART* APEXII CCD
Absorption correction	Multi-scan (*SADABS*; Bruker, 2008 ▸)
*T*_min_, *T*_max_	0.979, 0.986
No. of measured, independent and observed [*I* > 2σ(*I*)] reflections	43393, 5246, 3546
*R*_int_	0.028
(sin θ/λ)_max_ (Å^−1^)	0.707

Refinement	
*R*[*F* ^2^ > 2σ(*F* ^2^)], *wR*(*F* ^2^), *S*	0.053, 0.169, 1.02
No. of reflections	5246
No. of parameters	221
H-atom treatment	H-atom parameters constrained
Δρ_max_, Δρ_min_ (e Å^−3^)	0.45, −0.22

Computer programs: *APEX2* and *SAINT*, *SHELXS97* and *SHELXL97* (Sheldrick, 2008 ▸), *SHELXL2018* (Sheldrick, 2015 ▸), *ORTEP-3 for Windows* (Farrugia, 2012 ▸) and *PLATON* (Spek, 2020 ▸).

## supplementary crystallographic information

### Crystal data

**Table:**

C_22_H_27_NO	*F*(000) = 696
*M**_r_* = 321.44	*D*_x_ = 1.155 Mg m^−^^3^
Monoclinic, *P*2_1_/*n*	Mo *K*α radiation, λ = 0.71073 Å
*a* = 13.3077 (5) Å	Cell parameters from 3546 reflections
*b* = 10.3009 (4) Å	θ = 1.9–30.2°
*c* = 13.9338 (5) Å	µ = 0.07 mm^−^^1^
β = 104.657 (1)°	*T* = 296 K
*V* = 1847.91 (12) Å^3^	Block, white crystalline
*Z* = 4	0.30 × 0.25 × 0.20 mm

### Data collection

**Table:**

Bruker SMART APEXII CCD diffractometer	3546 reflections with *I* > 2σ(*I*)
Radiation source: fine-focus sealed tube	*R*_int_ = 0.028
ω and φ scans	θ_max_ = 30.2°, θ_min_ = 1.9°
Absorption correction: multi-scan (*SADABS*; Bruker, 2008)	*h* = −18→18
*T*_min_ = 0.979, *T*_max_ = 0.986	*k* = −14→14
43393 measured reflections	*l* = −19→19
5246 independent reflections	

### Refinement

**Table:**

Refinement on *F*^2^	Hydrogen site location: inferred from neighbouring sites
Least-squares matrix: full	H-atom parameters constrained
*R*[*F*^2^ > 2σ(*F*^2^)] = 0.053	*w* = 1/[σ^2^(*F*_o_^2^) + (0.0897*P*)^2^ + 0.2822*P*] where *P* = (*F*_o_^2^ + 2*F*_c_^2^)/3
*wR*(*F*^2^) = 0.169	(Δ/σ)_max_ = 0.001
*S* = 1.02	Δρ_max_ = 0.45 e Å^−^^3^
5246 reflections	Δρ_min_ = −0.22 e Å^−^^3^
221 parameters	Extinction correction: SHELXL2018 (Sheldrick, 2015), Fc^*^=kFc[1+0.001xFc^2^λ^3^/sin(2θ)]^-1/4^
0 restraints	Extinction coefficient: 0.028 (3)

### Special details

**Table:**

Geometry. All esds (except the esd in the dihedral angle between two l.s. planes) are estimated using the full covariance matrix. The cell esds are taken into account individually in the estimation of esds in distances, angles and torsion angles; correlations between esds in cell parameters are only used when they are defined by crystal symmetry. An approximate (isotropic) treatment of cell esds is used for estimating esds involving l.s. planes.

### Fractional atomic coordinates and isotropic or equivalent isotropic displacement parameters (Å^2^)

**Table:**

	*x*	*y*	*z*	*U*_iso_*/*U*_eq_	
C2	0.65890 (11)	−0.04974 (12)	0.67960 (9)	0.0430 (3)	
H2	0.710803	−0.117590	0.703575	0.052*	
C3	0.55791 (12)	−0.11907 (14)	0.63075 (11)	0.0535 (4)	
H3A	0.509789	−0.058442	0.589793	0.064*	
H3B	0.571443	−0.188289	0.588507	0.064*	
C4	0.51045 (13)	−0.17493 (14)	0.70987 (12)	0.0553 (4)	
H4A	0.562934	−0.221549	0.758707	0.066*	
H4B	0.455515	−0.235360	0.680048	0.066*	
C5	0.46657 (10)	−0.06466 (13)	0.75983 (10)	0.0434 (3)	
H5	0.406942	−0.029180	0.710452	0.052*	
C6	0.54640 (9)	0.04763 (12)	0.79099 (9)	0.0386 (3)	
H6	0.561919	0.050544	0.863560	0.046*	
C7	0.50324 (9)	0.18152 (12)	0.75655 (9)	0.0401 (3)	
C8	0.44865 (11)	0.20754 (15)	0.65996 (11)	0.0509 (3)	
H8	0.438587	0.141664	0.612841	0.061*	
C9	0.40861 (12)	0.33048 (16)	0.63217 (13)	0.0608 (4)	
H9	0.372408	0.346381	0.566876	0.073*	
C10	0.42249 (12)	0.42814 (15)	0.70093 (15)	0.0649 (5)	
H10	0.395238	0.510190	0.682456	0.078*	
C11	0.47650 (13)	0.40503 (15)	0.79693 (15)	0.0639 (4)	
H11	0.486050	0.471484	0.843544	0.077*	
C12	0.51698 (11)	0.28257 (14)	0.82473 (11)	0.0508 (3)	
H12	0.553878	0.267904	0.889987	0.061*	
C13	0.69896 (10)	0.03120 (13)	0.60592 (9)	0.0435 (3)	
C14	0.68956 (14)	0.16488 (15)	0.59909 (12)	0.0593 (4)	
H14	0.656620	0.209142	0.640704	0.071*	
C15	0.72856 (16)	0.23353 (17)	0.53114 (13)	0.0693 (5)	
H15	0.722127	0.323421	0.527699	0.083*	
C16	0.77685 (15)	0.16916 (19)	0.46859 (13)	0.0695 (5)	
H16	0.803555	0.215281	0.423235	0.083*	
C17	0.78528 (15)	0.03651 (18)	0.47370 (13)	0.0664 (5)	
H17	0.816965	−0.007417	0.430922	0.080*	
C18	0.74709 (12)	−0.03242 (15)	0.54185 (11)	0.0528 (4)	
H18	0.753688	−0.122304	0.544822	0.063*	
C19	0.73417 (10)	0.06355 (13)	0.83744 (10)	0.0443 (3)	
C20	0.83981 (11)	0.02750 (16)	0.82495 (13)	0.0565 (4)	
H20A	0.851320	0.070404	0.767506	0.085*	
H20B	0.843344	−0.064779	0.816729	0.085*	
H20C	0.892096	0.053889	0.882667	0.085*	
C21	0.42650 (12)	−0.10889 (15)	0.84920 (12)	0.0546 (4)	
H21	0.486985	−0.132684	0.902717	0.065*	
C22	0.37021 (14)	0.00100 (17)	0.88713 (14)	0.0665 (5)	
H22A	0.313140	0.030351	0.834665	0.100*	
H22B	0.417520	0.071598	0.909149	0.100*	
H22C	0.344502	−0.029708	0.941477	0.100*	
C23	0.35598 (17)	−0.22597 (19)	0.82723 (17)	0.0826 (6)	
H23A	0.299342	−0.208302	0.770673	0.124*	
H23B	0.329279	−0.244610	0.883608	0.124*	
H23C	0.394522	−0.299376	0.813439	0.124*	
N1	0.64819 (8)	0.02265 (10)	0.76798 (7)	0.0396 (2)	
O1	0.72737 (8)	0.12759 (12)	0.90987 (7)	0.0591 (3)	

### Atomic displacement parameters (Å^2^)

**Table:**

	*U*^11^	*U*^22^	*U*^33^	*U*^12^	*U*^13^	*U*^23^
C2	0.0512 (7)	0.0375 (6)	0.0451 (7)	0.0024 (5)	0.0209 (6)	−0.0028 (5)
C3	0.0679 (9)	0.0462 (7)	0.0528 (8)	−0.0114 (7)	0.0272 (7)	−0.0134 (6)
C4	0.0683 (9)	0.0406 (7)	0.0652 (9)	−0.0110 (6)	0.0322 (7)	−0.0093 (6)
C5	0.0452 (7)	0.0406 (6)	0.0475 (7)	−0.0036 (5)	0.0172 (5)	−0.0013 (5)
C6	0.0403 (6)	0.0406 (6)	0.0370 (6)	−0.0001 (5)	0.0135 (5)	−0.0022 (5)
C7	0.0361 (6)	0.0390 (6)	0.0482 (7)	−0.0005 (5)	0.0159 (5)	−0.0026 (5)
C8	0.0510 (8)	0.0485 (7)	0.0519 (8)	0.0004 (6)	0.0102 (6)	0.0004 (6)
C9	0.0479 (8)	0.0585 (9)	0.0731 (10)	0.0054 (7)	0.0097 (7)	0.0153 (8)
C10	0.0458 (8)	0.0446 (8)	0.1057 (14)	0.0073 (6)	0.0220 (9)	0.0102 (8)
C11	0.0551 (9)	0.0444 (8)	0.0961 (13)	0.0018 (6)	0.0264 (8)	−0.0164 (8)
C12	0.0490 (8)	0.0469 (7)	0.0585 (8)	−0.0001 (6)	0.0171 (6)	−0.0094 (6)
C13	0.0444 (7)	0.0455 (7)	0.0436 (7)	−0.0016 (5)	0.0166 (5)	−0.0024 (5)
C14	0.0767 (10)	0.0461 (8)	0.0656 (9)	0.0005 (7)	0.0375 (8)	0.0000 (7)
C15	0.0948 (13)	0.0505 (9)	0.0723 (10)	−0.0069 (8)	0.0390 (9)	0.0065 (8)
C16	0.0826 (12)	0.0743 (11)	0.0609 (9)	−0.0179 (9)	0.0357 (9)	0.0030 (8)
C17	0.0757 (11)	0.0735 (11)	0.0628 (9)	−0.0068 (9)	0.0411 (8)	−0.0087 (8)
C18	0.0575 (8)	0.0527 (8)	0.0548 (8)	0.0004 (6)	0.0264 (7)	−0.0062 (6)
C19	0.0435 (7)	0.0426 (7)	0.0472 (7)	0.0009 (5)	0.0122 (5)	0.0026 (5)
C20	0.0419 (7)	0.0572 (9)	0.0714 (10)	0.0005 (6)	0.0162 (7)	0.0017 (7)
C21	0.0575 (8)	0.0525 (8)	0.0612 (8)	−0.0021 (6)	0.0287 (7)	0.0055 (6)
C22	0.0701 (10)	0.0686 (10)	0.0743 (10)	−0.0087 (8)	0.0433 (9)	−0.0094 (8)
C23	0.0985 (15)	0.0604 (11)	0.1080 (15)	−0.0175 (10)	0.0617 (12)	−0.0009 (10)
N1	0.0411 (5)	0.0404 (5)	0.0398 (5)	0.0016 (4)	0.0149 (4)	−0.0026 (4)
O1	0.0509 (6)	0.0719 (7)	0.0518 (6)	−0.0013 (5)	0.0080 (4)	−0.0156 (5)

### Geometric parameters (Å, º)

**Table:**

C2—N1	1.4770 (15)	C13—C14	1.384 (2)
C2—C13	1.5199 (18)	C13—C18	1.3878 (18)
C2—C3	1.522 (2)	C14—C15	1.384 (2)
C2—H2	0.9800	C14—H14	0.9300
C3—C4	1.516 (2)	C15—C16	1.377 (3)
C3—H3A	0.9700	C15—H15	0.9300
C3—H3B	0.9700	C16—C17	1.371 (3)
C4—C5	1.5238 (19)	C16—H16	0.9300
C4—H4A	0.9700	C17—C18	1.381 (2)
C4—H4B	0.9700	C17—H17	0.9300
C5—C21	1.5422 (19)	C18—H18	0.9300
C5—C6	1.5561 (18)	C19—O1	1.2280 (16)
C5—H5	0.9800	C19—N1	1.3648 (17)
C6—N1	1.4913 (15)	C19—C20	1.5061 (19)
C6—C7	1.5241 (17)	C20—H20A	0.9600
C6—H6	0.9800	C20—H20B	0.9600
C7—C8	1.3843 (19)	C20—H20C	0.9600
C7—C12	1.3897 (18)	C21—C23	1.511 (2)
C8—C9	1.390 (2)	C21—C22	1.524 (2)
C8—H8	0.9300	C21—H21	0.9800
C9—C10	1.369 (2)	C22—H22A	0.9600
C9—H9	0.9300	C22—H22B	0.9600
C10—C11	1.370 (3)	C22—H22C	0.9600
C10—H10	0.9300	C23—H23A	0.9600
C11—C12	1.388 (2)	C23—H23B	0.9600
C11—H11	0.9300	C23—H23C	0.9600
C12—H12	0.9300		
			
N1—C2—C13	114.24 (10)	C14—C13—C18	118.30 (13)
N1—C2—C3	110.44 (10)	C14—C13—C2	123.46 (12)
C13—C2—C3	112.10 (11)	C18—C13—C2	118.25 (12)
N1—C2—H2	106.5	C15—C14—C13	120.84 (15)
C13—C2—H2	106.5	C15—C14—H14	119.6
C3—C2—H2	106.5	C13—C14—H14	119.6
C4—C3—C2	109.64 (12)	C16—C15—C14	120.20 (16)
C4—C3—H3A	109.7	C16—C15—H15	119.9
C2—C3—H3A	109.7	C14—C15—H15	119.9
C4—C3—H3B	109.7	C17—C16—C15	119.48 (15)
C2—C3—H3B	109.7	C17—C16—H16	120.3
H3A—C3—H3B	108.2	C15—C16—H16	120.3
C3—C4—C5	109.13 (11)	C16—C17—C18	120.55 (15)
C3—C4—H4A	109.9	C16—C17—H17	119.7
C5—C4—H4A	109.9	C18—C17—H17	119.7
C3—C4—H4B	109.9	C17—C18—C13	120.63 (15)
C5—C4—H4B	109.9	C17—C18—H18	119.7
H4A—C4—H4B	108.3	C13—C18—H18	119.7
C4—C5—C21	113.54 (11)	O1—C19—N1	121.72 (12)
C4—C5—C6	111.60 (11)	O1—C19—C20	119.50 (13)
C21—C5—C6	110.21 (11)	N1—C19—C20	118.78 (12)
C4—C5—H5	107.0	C19—C20—H20A	109.5
C21—C5—H5	107.0	C19—C20—H20B	109.5
C6—C5—H5	107.0	H20A—C20—H20B	109.5
N1—C6—C7	112.27 (10)	C19—C20—H20C	109.5
N1—C6—C5	113.89 (10)	H20A—C20—H20C	109.5
C7—C6—C5	114.11 (10)	H20B—C20—H20C	109.5
N1—C6—H6	105.2	C23—C21—C22	109.18 (14)
C7—C6—H6	105.2	C23—C21—C5	113.34 (13)
C5—C6—H6	105.2	C22—C21—C5	111.18 (13)
C8—C7—C12	117.74 (13)	C23—C21—H21	107.6
C8—C7—C6	122.99 (12)	C22—C21—H21	107.6
C12—C7—C6	119.25 (12)	C5—C21—H21	107.6
C7—C8—C9	121.13 (14)	C21—C22—H22A	109.5
C7—C8—H8	119.4	C21—C22—H22B	109.5
C9—C8—H8	119.4	H22A—C22—H22B	109.5
C10—C9—C8	120.00 (15)	C21—C22—H22C	109.5
C10—C9—H9	120.0	H22A—C22—H22C	109.5
C8—C9—H9	120.0	H22B—C22—H22C	109.5
C9—C10—C11	120.01 (15)	C21—C23—H23A	109.5
C9—C10—H10	120.0	C21—C23—H23B	109.5
C11—C10—H10	120.0	H23A—C23—H23B	109.5
C10—C11—C12	120.09 (15)	C21—C23—H23C	109.5
C10—C11—H11	120.0	H23A—C23—H23C	109.5
C12—C11—H11	120.0	H23B—C23—H23C	109.5
C11—C12—C7	121.02 (15)	C19—N1—C2	120.44 (11)
C11—C12—H12	119.5	C19—N1—C6	116.00 (10)
C7—C12—H12	119.5	C2—N1—C6	123.43 (10)
			
N1—C2—C3—C4	−39.81 (16)	C18—C13—C14—C15	−1.0 (3)
C13—C2—C3—C4	−168.43 (12)	C2—C13—C14—C15	179.32 (15)
C2—C3—C4—C5	72.53 (16)	C13—C14—C15—C16	0.5 (3)
C3—C4—C5—C21	−173.93 (13)	C14—C15—C16—C17	0.5 (3)
C3—C4—C5—C6	−48.63 (16)	C15—C16—C17—C18	−0.9 (3)
C4—C5—C6—N1	−1.38 (15)	C16—C17—C18—C13	0.4 (3)
C21—C5—C6—N1	125.74 (12)	C14—C13—C18—C17	0.5 (2)
C4—C5—C6—C7	129.33 (12)	C2—C13—C18—C17	−179.74 (14)
C21—C5—C6—C7	−103.55 (13)	C4—C5—C21—C23	−48.70 (19)
N1—C6—C7—C8	82.58 (15)	C6—C5—C21—C23	−174.73 (14)
C5—C6—C7—C8	−48.92 (16)	C4—C5—C21—C22	−172.13 (14)
N1—C6—C7—C12	−98.65 (13)	C6—C5—C21—C22	61.84 (16)
C5—C6—C7—C12	129.85 (12)	O1—C19—N1—C2	177.54 (12)
C12—C7—C8—C9	−0.2 (2)	C20—C19—N1—C2	−3.16 (18)
C6—C7—C8—C9	178.56 (13)	O1—C19—N1—C6	−6.49 (18)
C7—C8—C9—C10	−0.3 (2)	C20—C19—N1—C6	172.81 (11)
C8—C9—C10—C11	0.5 (3)	C13—C2—N1—C19	−69.70 (15)
C9—C10—C11—C12	−0.2 (2)	C3—C2—N1—C19	162.84 (12)
C10—C11—C12—C7	−0.4 (2)	C13—C2—N1—C6	114.64 (13)
C8—C7—C12—C11	0.6 (2)	C3—C2—N1—C6	−12.81 (17)
C6—C7—C12—C11	−178.28 (13)	C7—C6—N1—C19	87.19 (13)
N1—C2—C13—C14	−23.54 (19)	C5—C6—N1—C19	−141.19 (11)
C3—C2—C13—C14	103.05 (16)	C7—C6—N1—C2	−96.97 (13)
N1—C2—C13—C18	156.75 (12)	C5—C6—N1—C2	34.64 (15)
C3—C2—C13—C18	−76.66 (16)		

### Hydrogen-bond geometry (Å, º)

**Table:**

*D*—H···*A*	*D*—H	H···*A*	*D*···*A*	*D*—H···*A*
C9—H9···O1^i^	0.93	2.54	3.4378 (19)	163

Symmetry code: (i) *x*−1/2, −*y*+1/2, *z*−1/2.
